# Five markers useful for the distinction of canine mammary malignancy

**DOI:** 10.1186/1746-6148-9-138

**Published:** 2013-07-11

**Authors:** Karol M Pawłowski, Henryk Maciejewski, Kinga Majchrzak, Izabella Dolka, Jan A Mol, Tomasz Motyl, Magdalena Król

**Affiliations:** 1 Department of Physiological Sciences, Faculty of Veterinary Medicine, Warsaw University of Life Sciences - WULS, Nowoursynowska 159, 02-776, Warsaw, Poland; 2 Department of Large Animal Diseases with Clinic, Faculty of Veterinary Medicine, Warsaw University of Life Sciences – WULS, Nowoursynowska 100, 02-797, Warsaw, Poland; 3 Institute of Computer Engineering, Control and Robotics I-6, Wroclaw University of Technology, Wybrzeże Wyspiańskiego 27, 50-320, Wroclaw, Poland; 4 Department of Animal Environment Biology, Faculty of Animal Sciences, Warsaw University of Life Sciences - WULS, Ciszewskiego 8, 02-786, Warsaw, Poland; 5 Department of Pathology and Veterinary Diagnostics, Faculty of Veterinary Medicine, Warsaw University of Life Sciences - WULS, Nowoursynowska 159, 02-776, Warsaw, Poland; 6 Department Clinical Sciences of Companion Animals, Faculty of Veterinary Medicine, Utrecht University, Yalelaan 108, 3584 CM, Utrecht, The Netherlands

**Keywords:** Canine mammary malignancy, Malignancy classifier, sehrl, mipep, magi3, zfp37, Relaxin

## Abstract

**Background:**

Spontaneous canine mammary tumors constitute a serious clinical problem. There are significant differences in survival between cases with different tumor grades. Unfortunately, the distinction between various grades is not clear. A major problem in evaluating canine mammary cancer is identifying those, that are “truly” malignant. That is why the aim of our study was to find the new markers of canine malignancy, which could help to diagnose the most malignant tumors.

**Results:**

Analysis of gene expression profiles of canine mammary carcinoma of various grade of malignancy followed by the boosted tree analysis distinguished a `gene set`. The expression of this gene set (*sehrl*, *zfp37*, *mipep*, *relaxin*, and *magi3*) differs significantly in the most malignant tumors at mRNA level as well as at protein level. Despite this `gene set` is very interesting as an additional tool to estimate canine mammary malignancy, it should be validated using higher number of samples.

**Conclusions:**

The proposed gene set can constitute a `malignancy marker` that could help to distinguish the most malignant canine mammary carcinomas. These genes are also interesting as targets for further investigations and therapy. So far, only two of them were linked with the cancer development.

## Background

Spontaneous mammary tumors are the most common type of cancer in women and in female dogs. The annual incidence in dogs is 3 times more frequent than in humans. Thus, it constitutes a serious clinical problem [1]. Unfortunately, the distinction between various grades of tumors can be considerably less clear than one would like [2]. A major problem in evaluating canine mammary cancer is identifying those, that are “truly” malignant [3]. The most significant criteria for the diagnosis of malignant mammary tumors in the dog are as follow: tumor type, significant nuclear and cellular pleomorphism, mitotic index, presence of randomly distributed areas of necrosis within the neoplasm, peritumoral and lymphatic invasion, and regional lymph node metastasis. However, classification based on these criteria sometimes leads to the over-diagnosis of mammary carcinoma [3]. However, good pathological diagnosis constitute basis to yet proper prognosis [1]. Karayannopoulou et al. [4] found significant differences in survival between cases with different tumor grades: survival was worse in dogs with grade III carcinomas than in those with grade I or grade II.

The advent of DNA microarrays - a modern and powerful tool for investigation of biological processes through the widespread analysis of genes from a particular cell, tissue, or organism - enabled investigators to identify characteristic expression patterns of groups of genes that are associated with specific tumor traits. They are a great hope of finding new cancer markers, markers of clinical outcome, and targets for anticancer therapy [5].

The purpose of this preliminary study was to point out find new markers of canine mammary malignancy (that could help to improve pathological diagnosis of the tumor grade) based on the gene expression. We built the molecular classifier, which was able to distinguish the most malignant canine mammary tumors from the lowest estimated malignant tumors. As of the highest grade of malignancy is associated with an increased risk of death within 2 years after mastectomy [4], proper diagnosis may lead to prediction to the clinical outcome.

## Methods

### Tissue samples

Tumor samples of canine mammary cancers were obtained from patients subjected to surgery. The tumors then, were divided into two halves, one of them was fixed in 10% neutral buffered formalin and routinely embedded in paraffin to perform histological assay (n = 78 samples in total). Whereas the other one was snap frozen in liquid nitrogen and stored in −80°C (n = 18). Four μm samples from paraffin blocks were fixed on glass slides, stained with haematoxylin – eosin (HE) and had been examined by certified pathologists (prof. Dr. hab. Elżbieta Malicka and Dr. Izabella Dolka, both from the Warsaw University of Life Sciences, Poland). The immunohistochemical examination of cytokeratin, vimentin, smooth muscle actin, s100 protein and p63 protein expression was performed in order to diagnose these tumors (data not shown). The tumor types of specimens were classified based on the World Health Organization (WHO) Histological Classification and Mammary Tumors of the Dog and Cat classification [6]. Histological tumor grading was conducted on HE-stained sections using a Misdorp classification [6]. The mammary carcinoma grading was assessed in respect to tubule formation, degree of differentiation and mitotic index as grade I, grade II, or grade III tumor. The tumors dedicated to this study were non-metastatic (no metastasis detected in lymph nodes or lungs of the patients). Clinical information about the outcome of these patients is unknown.

### Statistical analysis and building of the classifier of canine mammary malignancy

**Figure 1:**
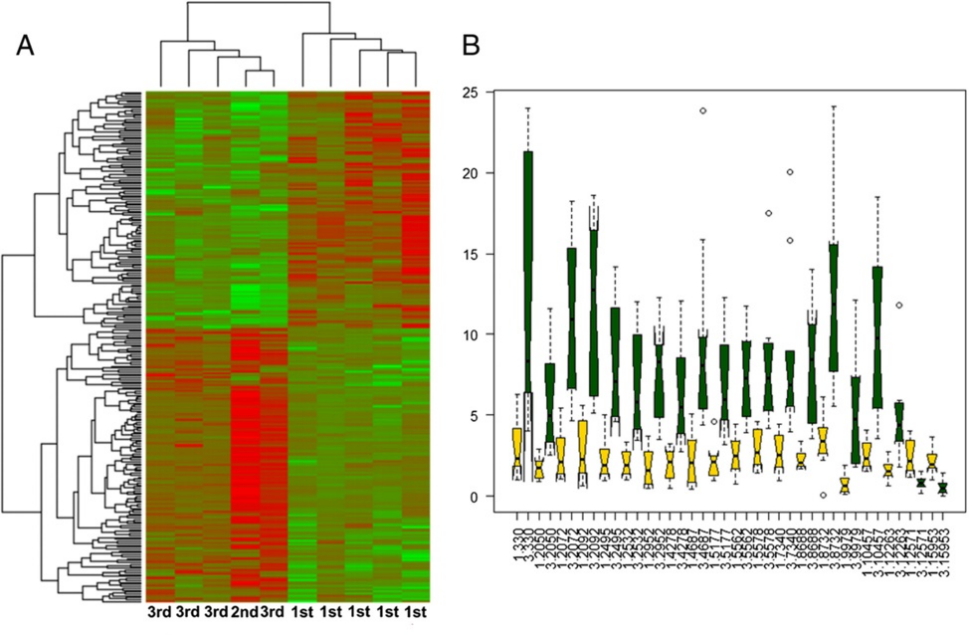
**The clustering of tumors taken to the analysis and variation in expression of 20 the mostly significant genes between the most and less malignant canine mammary tumors. A.** Differences in expression of genes in 12 examined samples. Data is presented in a matrix format: each row represents a single gene, and each column an experimental sample. In each sample, the ratio of the abundance of transcripts of each gene to the median abundance of the gene's transcript across all tissue samples is represented by the color of the corresponding cell in the matrix. Green squares, transcript levels below the median; red squares, transcript levels greater than the median. Color saturation reflects the magnitude of the ratio relative to the median for each set of samples. **B.** Boxplots comparing relative signal measured for 20 most significantly changed genes between less malignant (yellow) and the most malignant (green) tumors.

To create the molecular classifier of canine mammary malignancy we used our previous data [1] deposited in NCBI's Gene Expression Omnibus (GEO) with Series accession number GSE 29601. Briefly, the statistical analysis of gene expression was performed using linear methods for microarrays (limma package in Bioconductor software) [7]. The method tests the null hypothesis of no differential expression between the groups of samples compared was performed by using the moderated t-statistic [7], which has similar interpretation as the ordinary t-test statistic. The expression of genes with the Benjamini-Hochberg (FDR) multiple-testing corrected p-value below 0.05 was qualified as significantly changed. Each of the microarrays had been examined separately. Our previously conducted unsupervised analysis [1] had classified the examined canine mammary tumor tissues (grade I, grade II and grade III) into three groups. For the purposes of the hereby study we were mainly focused on two of them: the 1^st^ which consisted of 4 grade III and 1 grade II carcinomas (it was the most malignant group) and the second consisted of 4 grade I carcinomas (it was the group consisted of the lowest estimated malignant tumors) (Figure 1A). We were focused only on these two groups to find genes that present exact characteristic for the most malignant tumors. Thus, to build a canine mammary malignancy classifier the following training data were used: the most malignant tumors (n = 8, two hybridizations per patient) and the lowest estimated malignant tumors (n = 10, two hybridizations per patient).

We removed genes with low expression (arbitrary threshold was used where genes with signal < 100 in more than half of the samples tested were removed). First, we identified 20 most differentially expressed genes (Table 1, Figure 1B) between the groups of the most malignant and the lowest estimated malignant tumors in the training data. Analysis of differentially expressed genes was done based on the nonparametric Wilcoxon test. These genes were used as features for training the classifier of canine mammary the most malignant tumors.

**Table 1:** Twenty the highly significant genes in the most malignant tumors

**pValue**	**Gene ID**
4.570593e-05	DG9-150a24
4.570593e-05	DG11-200c22
9.141186e-05	DG11-134o13
9.141186e-05	DG9-26a1
9.141186e-05	DG9-122e24
1.828237e-04	DG9-43e14
1.828237e-04	DG9-230a23
1.828237e-04	DG9-230c14
1.828237e-04	DG9-149d7
1.828237e-04	DG9-94o23
1.828237e-04	DG11-126 g2
1.828237e-04	DG2-37 k21
1.828237e-04	DG9-44 k24
1.828237e-04	DG14-3 m8
1.828237e-04	DG9-88 k19
1.828237e-04	DG14-10o2
1.828237e-04	DG32-60e11
1.828237e-04	DG8-30 l15
3.199415e-04	DG11-180e10
3.199415e-04	DG8-183d10

Twenty genes that were significantly changed in grade III tumors. This gene set was selected based on the calculation of the gene expression of all the spots from all microarrays (n = 36) versus median expression of the reference genes for canine mammary cancer [7,8] hybridized on the same microarrays: *rps19*, *cgi*-*119*, *ctbp1* and *b2m*.

Prior to building the classifier we calculated expression of the selected genes relative to the median expression the reference genes as measured on the same microarray (*rps19*, *cgi*-*119*, *ctbp1* and *b2m* were used as the reference genes) [8,9]. We built the classifier using this relative signal calculated for features listed in Table 1. The decision tree classifier was built with the use of boosted C5.0 algorithm.

The gene function was identified with the use of NCBI database and PANTHER pathway analysis software [9]. The pathway analyses were conducted using one-way ANOVA with binominal Bonferroni statistic test (PANTHER) with the cut-off value p < 0.05.

### Real-time qPCR

The mRNA sequences of the key genes were obtained from NCBI database. Primers were designed with the PRIMER3 software (free on-line access) and checked using Oligo Calculator (free on-line access) and Primer-Blast (NCBI database). Primers’ sequences are listed in Table 2. *hprt* and *rps19* genes were used as non-regulated reference genes for normalization of target gene expression [8,9]. For the analysis, n = 18 canine mammary cancer samples were used (6 tumors of each grade). Quantitative RT-PCR was performed with the fluorogenic Lightcycler Fast Strand DNA Syber Green (Roche) and the Light Cycler (Roche). The results were analyzed based on comparative Ct method [10]. Relative transcript abundance of the gene equals ΔCt values (ΔCt = Ct^reference^ – Ct^target^). Relative changes in transcript are expressed as ΔΔCt values (ΔΔCt = ΔCt^examined sample^ – ΔCt^control^). The experiment was conducted three times.

**Table 2:** Primers used for real-time qPCR

**Gene symbol**	**Forward primer**	**Reverse primer**	**Optimum annealing temp. (°C)**	**Optimum annealing time (sec)**
*hprt*	AGCTTGCTGGTGAAAAGGAC	TTATAGTCAAGGGCATATCC	59	6
*rps19*	CCTTCCTCAAAAAGTCTGGG	GTTCTCATCGTAGGGAGCAAG	61	10
*serhl*	TTGGTGCTAGACACGCTGAG	CTGCAGAAAGGCACTGATGA	61	5
*zfp37*	GCGGAATGGGAACAACTAGA	ATGTCTGGTTTGGGAGCTTG	61	8
*mipep*	CCCTGAGAAAGGCAGACTTG	AGCCACTCTGCACAAGGAAT	60	5
*relaxin*	AAGTTGTGCCATCCTCCATC	CCAGACCGTGTTGCTATCCT	60	7
*magi3*	AGGTGACATTGGGAAAGACG	CAGCCCCATGTTGTACTCCT	60	8

Primers sequences used in this study and their annealing optimal temperature and time. The mRNA sequences of key genes were obtained from NCBI database. Primers were designed using PRIMER3 software (free on-line access) and checked using Oligo Calculator (free on-line access) and Primer-Blast (NCBI database). *hprt* and *rps19* genes were used as non-regulated reference genes for normalization of target gene expression [6,7].

### Immunohistochemistry

Four μm sections from paraffin blocks containing tumour tissue (n = 60 in total; 20 tumor tissues from each grade of malignancy) were baked in 37°C overnight. After dewaxing in xylene and rehydration in ethanol, for antigen retrieval, the slides were placed in 0.02 M citrate buffer, pH 6.0 and boiled in the decloaking chamber. The samples were incubated in the Peroxidase Blocking Reagent (Dako, Denmark) for 10 min at room temperature prior to the antibody incubation. After 30 min incubation in 5% bovine serum albumin (Sigma Aldrich, Germany), the following mouse/rabbit primary antibodies were used (diluted 1:100 in 1% bovine serum): anti MAGI3, ZFP37, SERHL, Relaxin, MIPEP (all of them obtained from Abcam, UK). The slides were incubated with antibodies overnight at +4°C. For the staining the EnVision kit (Dako) was used (Labelled Polymers consist of secondary anti-mouse or anti-rabbit antibodies conjugated with the Horseradish peroxidase HRP enzyme complex). To develop the colored product, the 3,3`-Diaminobenzidine (DAB) substrate was used. Finally, the haematoxylin was taken for nuclei counterstaining. To confirm the differences in expression of these antigens between tumors of the various grade, n = 60 samples per each antibody were examined.

For each immunohistochemical experiment the negative control was used (the staining without the use of primary antibodies).

### Statistical analysis

The statistical analysis of Real-time qPCR and immunohistochemistry was conducted using Prism version 5.00 software (GraphPad Software, USA). The one-way ANOVA, and ANOVA + Tukey HSD (Honestly Significant Difference) post-hoc test were applied. The p-value <0.05 was regarded as significant whereas p-value <0.01 and p-value <0.001 as highly significant.

## Results

### Selection of gene set used for diagnosis of malignant tumor

In the first analysis we built the classifier based on the training data. The algorithm selected five genes: *mipep*, *serhl*, *relaxin*, *magi3* and *zfp37* which expression can allow us to diagnose the most malignant tumors. The boosted tree rules were as follow:

Rule 1. estimated accuracy 95.65% [boost 100%]

sehrl < = 4.046 [ Mode: 1 ] = > 1

sehrl > 4.046 [ Mode: 3 ] = > 3

Rule 2. estimated accuracy 96.17% [boost 100%]

zfp37 < = 3.377 [ Mode: 1 ] = > 3

zfp37 > 3.377 [ Mode: 3 ] = > 1

Rule 3. estimated accuracy 96.57% [boost 100%]

zfp37 < = 2.914 [ Mode: 1 ] = > 3

zfp37 > 2.914 [ Mode: 3 ] = > 1

Rule 4. estimated accuracy 95.48% [boost 100%]

mipep < = 1.257 [ Mode: 1 ] = > 1

mipep > 1.257 [ Mode: 3 ] = > 3

Rule 5. estimated accuracy 88.44% [boost 100%]

magi3 < = 1.458 [ Mode: 3 ] = > 3

magi3 > 1.458 [ Mode: 1 ] = > 1

Rule 6. estimated accuracy 100% [boost 100%]

sehrl < = 4.046 [ Mode: 1 ]

relaxin1 < = 4.203 [ Mode: 1 ] = > 1

relaxin1 > 4.203 [ Mode: 3 ] = > 3

sehrl > 4.046 [ Mode: 3 ] = > 3

Its efficacy assessed using the learning data was 100% in case of the most malignant tumors.

### Confirmation at mRNA level of the expression patterns of `diagnostic` set of genes in canine mammary carcinomas of various grade of malignancy

**Figure 2:**
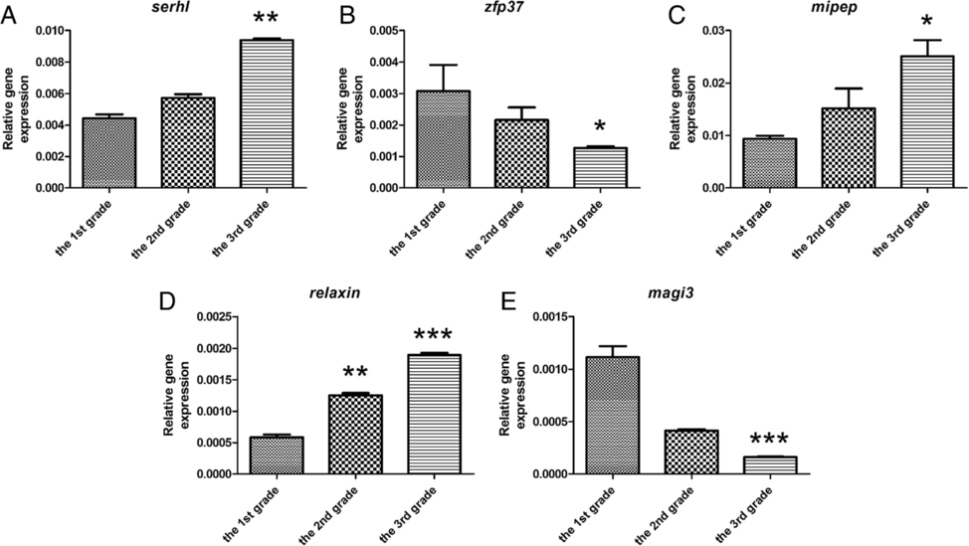
**Expression of `diagnostic gene set` assessed using real-time qPCR.** Expression of `diagnostic gene set` in canine mammary carcinoma of various grade of malignancy. The changes in gene expression which differed significantly (p < 0.05) markered as *, the changes in gene expression which differed highly significant (p < 0.01 and p < 0.001) markered as ** and ***, respectively.

Real-Time qPCR analysis confirmed similar trends in expression of the examined five genes (Figure 2). To check the expression of the selected `gene set` in mammary tumors in general, all the 18 tumors were under analysis using microarrays [1]. These tumors were classified by pathologists as grade I, grade II and grade III (six of each group).

The highest relative expression of *serhl* showed grade III tumors (0.0095 ± 9.75e-005), whereas the lowest expression showed grade I tumors (0.0044 ± 0.0002). The relative mean expression of *serhl* in grade II tumors was 0.0057 (±0.0002). The relative expression of *serhl* differed highly significant between the most malignant tumors and other groups (p < 0.01) (Figure 2A).

The highest relative expression of *zfp37* showed grade I tumors (0.0031 ± 0.0008), whereas the lowest expression exposed the most malignant tumors (0.0012 ± 5.5e-005). The relative mean expression of *zfp37* in grade II tumors was 0.0022 (±0.0004). The relative expression of *zfp37* differed significantly between grade III tumors and others (p < 0.05) (Figure 2B).

The highest relative expression of *mipep* presented grade III tumors (0.025 ± 0.0044), whereas the lowest expression showed grade I tumors (0.009 ± 0.0005). The relative mean expression of *mipep* in grade II tumors was 0.015 (±0.0038). The relative expression of *mipep* differed significantly between most malignant tumors and others (p < 0.05) (Figure 2C).

The highest relative expression of *relaxin* gave us the result grade III tumors (0.0019 ± 3.25e-005), whereas the lowest expression showed grade I tumors (0.0006 ± 4.65e-005). The relative mean expression of *relaxin* in grade II tumors was 0.0013 (±3.5e-005). The relative expression of *relaxin* differed highly significant between grade I and grade III tumors (p < 0.001) and between grade I and grade II tumors as well as grade II and grade III tumors (p < 0.01) (Figure 2D).

The highest relative expression of *magi3* showed grade I tumors (0.0011 ± 0.0001), whereas the lowest expression was revealed in grade III tumors (0.0002 ± 5.5e-006). The relative mean expression of *magi3* in grade II tumors was 0.0004 (±1.3e-005). The relative expression of *magi3* differed significantly between grade III tumors and others (p < 0.001) (Figure 2E).

### Expression of selected set of markers at protein level

**Figure 3:**
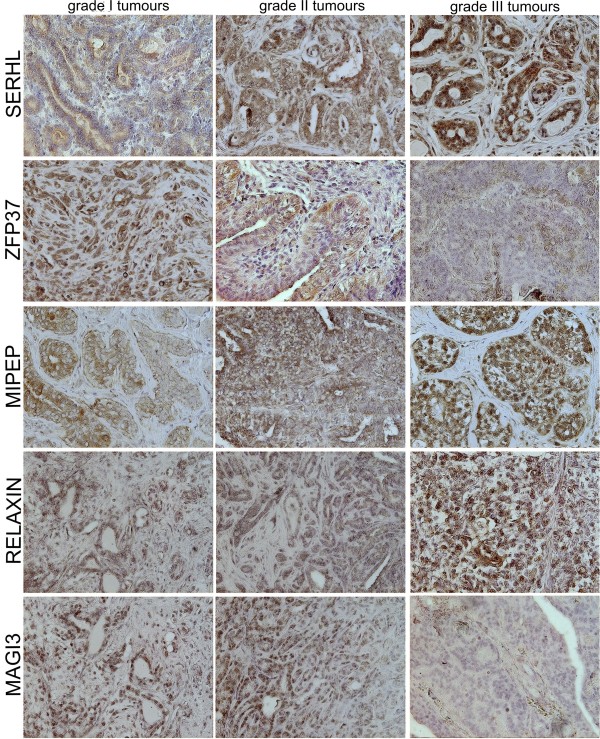
**Expression of proteins encoded by `diagnostic gene set`.** Pictures of SERHL, ZFP37, MIPEP, relaxin and MAGI3 in grade I, grade II and grade III canine mammary carcinomas (n = 60) obtained using Olympus BX60 microscope (x200). The examined antigen is presented as a brown precipitate. The colorimetric intensity of the IHC-stained antigen spots was counted by a computer-assisted image analyzer (Olympus Microimage™ Image Analysis, software version 4.0 for Windows, USA) and the antigen spot color intensity is expressed as mean pixel optical density on a 1–256 scale (data presented in Table 3).

Expression level of the proteins encoded by the `selected gene set`: SERHL, ZFP37, MIPEP, relaxin and MAGI3 was under examination by immunohistochemistry (Figure 3) in order to validate our previous findings. We examined 60 tumors (20 of each grade of malignancy). These tumors were classified by pathologists as: grade I, grade II or grade III. They were simple or complex carcinomas. No correlation between their type and level of proteins expression had been found.

The antibodies (described in Materials and Methods section) were dedicated to be used in various species, indicating their wide cross-specificity. However, we have performed the analysis of protein sequence in species which specificity was guaranteed by manufacturer and in canine by blasting them using NCBI tool. Additionally, we used the BLOSUM62 amino acid substitution matrix to score alignments between evolutionarily divergent protein sequences. Our results revealed a high similarity between these proteins in dogs and reactivity species, and thus based on the results we came up to use these antibodies in our experiments. The analysis of whole MAGI3 protein sequence showed 100% identity (protein accession numbers: Q9JK71 and J9P535), 94% identity in MIPEP (protein accession numbers: Q99797 and F1PWG7), 80% identity in ZFP37 (protein accession numbers: Q9Y6Q3 and J9P9T4), 64% identity in SERHL (protein accession numbers: Q9EPB5) and 41% identity in relaxin (protein accession numbers: P04808 and Q9TRM8). The identity of the epitope recognized by the antibodies or the peptide fragment used for immunization in the antibody`s manufacturing process was significant. We used similar approach in our previously published study [11].

We observed significantly (p < 0.01) increased expression of SERHL, MIPEP and relaxin in the grade III tumors comparing to the other tumors. SERHL expression in grade III tumors was 126.4 (±2.84), whereas in grade II and grade I tumors 107.8 (±4.10) and 109.2 (±2.99), respectively. MIPEP expression in grade III tumors was 123.5 (±3.88), whereas in grade II and grade I tumors it came up as 118.4 (±3.77) and 100.1 (±4.72), respectively. Expression of relaxin in grade III tumors was 125.2 (±1.08), whereas in grade II and grade I tumors it was 108.3 (±2.09) and 116.4 (±2.53). MIPEP expression was also significantly increased (p < 0.05) in grade II tumors comparing to the grade I. We also showed significantly (p < 0.05) and highly significant (p < 0.001) decreased expression of MAGI3 and ZFP37 (respectively) in the most malignant tumors (Table 3). Expression of MAGI3 in high-grade tumors was 103.0 (±1.87), whereas in grade II or grade I tumors it turned out to be 122.0 (±1.28) and 124.2 (±0.69). Expression of ZFP37 in grade III tumors was 103.6 (±3.13) whereas in grade II and grade I it was 122.8 (±2.70) and 124.2 (±1.46), respectively.

**Table 3:** Optical density related with the expression of examined proteins in canine mammary carcinoma at various grade of malignancy

**Antigen**	**Grade I tumors**	**Grade II tumors**	**Grade III tumors**
SERHL	109.2 (±2.99)	107.8 (±4.10)	126.4 (±2.84)**
ZFP37	124.2 (±1.46)	122.8 (2.70)	103.6 (3.13)***
MIPEP	100.1 (±4.72)	118.4 (±3.77)*	123.5 (±3.88)**
RELAXIN	116.4 (±2.53)	108.3 (2.09)	125.2 (1.08)**
MAGI3	124.2 (±0.69)	122.0 (±1.28)	103.0 (±1.87) *

The mean optical density of (±SD) related with expression of examined antigens assessed in Microimage software (Olympus). Mean values were calculated for 20 tumors of each pathological malignancy. For statistical purposes, the ANOVA + Tukey post-hoc tests were applied (Graph Pad Prism 5.0). The values that differed significantly (p < 0.05) markered as *, whereas the values that differed highly significant (p < 0.01 and p < 0.001) markered as ** and ***, respectively.

The mean optical density of the brown precipitates related to the examined antigen expression was showed in Table 3.

## Discussion

Analysis of gene expression profiles of canine mammary carcinoma of various grade of malignancy (n = 18) followed by the boosted tree analysis distinguished a `gene set` that might be helpful in diagnosis of canine mammary malignancy. This `set` consisted of five genes which expression have significantly changed in the most malignant tumors: *sehrl*, *zfp37*, *mipep*, *relaxin*, and *magi3*. Thus, based on their expression pattern the pathologist could distinguish between grade III tumors as well as others. In case of canine mammary tumors, the proper diagnosis is very important because it constitutes basis to the prognosis. The increased histological grade of malignancy is associated with an increased risk of death within 2 years after mastectomy [4]. Despite the proposed `gene set` seems to be useful in malignancy diagnosis, its usefulness should be validated with higher number of samples. So far, these genes and their products have not been treated as `malignancy markers` in breast cancer. Due to similar epidemiological and clinical characteristics of such tumors in these both species, similar environmental conditions, epithelial nature of these tumors, and similar composition of tumor microenvironment, the use of canine mammary tumors as an experimental model for human breast cancers is relevant [11]. Thus, in our opinion these results could be assessed in humans in order to validate the influence of these genes on cancer malignant transformation. So far, only two of these five genes were linked to cancer. These genes have not yet been described as malignancy markers in humans. In our opinion this `gene set` could be also a interesting target for further investigation.

An increased expression of SERHL was observed during passive stretch of skeletal muscles. It is suggested that its up-regulation can be related with skeletal muscle growth in response to mechanical stimuli [12]. Similar conditions (stretching and mechanical stimuli) are observed in fast growing tumors. As being recognized the most malignant tumors are significantly larger than less malignant or benign tumors [13], increased expression of SEHRL in the grade III tumors is not surprising (Figure 2, Figure 3, Table 3).

The protein encoded by *zfp37* belongs to the huge family of zinc finger proteins, which are regulators of transcription. High ZFP37 expression was shown during neurons and chondrocyte development, however in adults it was detected only in brain and testes [14,15]. ZFP37 is supposed to take part in development and differentiation of the cell. The results of the presented study showed significantly decreased expression of this gene and its protein in the most malignant tumors (Figure 2, Figure 3, Table 3). It can indicate the role of ZFP37 in cancer differentiation due to decrease in differentiation during the malignant transformation process in cancer [16].

The other selected gene is *mipep* which is involved in cellular metabolism. The product of this gene performs the final step in processing of nuclear-encoded proteins targeted to the mitochondrial matrix or inner membrane [17]. This gene may contribute to the functional effects of frataxin deficiency and the clinical manifestations of Friedreich ataxia (FA). The exact function of frataxin is still unclear, however it has been implicated in iron homeostasis, protection from oxidative stress and apoptosis [18]. Conflicting results have been reported regarding the role of frataxin in cellular growth: both frataxin knockdown and frataxin over-expression impair cell growth. Despite the role of frataxin in cancer is unknown, the relation between FA and malignancy has been suggested [19].

The next gene which increased expression was characteristic for the most malignant canine mammary tumors was relaxin. This peptide hormone, which is known as an inhibitor of uterine contractility and promoter of lengthen of the interpubic ligament before partition [20], promotes growth and development of mammary gland [21,22]. Moreover, it increases expression and catalytic activities of matrix metalloproteinase (MMP) [20]. It has been also found to enhance invasiveness of cancer cells by MMPs modulation [20]. The results of the present study showed up-regulation of relaxin in the most malignant tumors (Figure 2, Figure 3, Table 3) which are usually the most invasive [23].

The last significant gene for canine mammary malignancy is *magi3*. The MAGI protein is usually detected in the tight junctions between cells [24]. This protein reacts with LPA2 receptors which expression is increased in tumors and during inflammation [25,26], but inflammation often accompanies cancer development [27,28]. Moreover, in normal cells the activation of this receptor is not required [29]. LPA2 can be activated by MAGI3 or NHERF-2. Knockdown of MAGI3 increases expression of NHERF-2, which enhances cell growth, COX-2 expression and ensures cancer resistance to chemotherapy [30,31]. These data correlates with our own findings that MAGI3 expression was significantly down regulated in the most malignant tumors (Figure 2, Figure 3, Table 3).

A group of Prof. Klopfleisch published a very interesting paper exposing presentation of changes in proteome at different stages of canine mammary cancer progression [32]. They found 48 proteins which expression significantly changed between benign and malignant tumors. These proteins do not overlap with our findings. Differences may be related with the fact that a group of Prof. Klopfleisch examined general differences between benign and malignant tumors, whereas we have analyzed specific differences between various grades of canine mammary malignancy. However, both studies showed that tumor progression is associated with a stepwise change in protein expression levels, not only between benign and malignant tumors, but also in a relation between less and more malignant ones.

## Conclusions

The genes given above can altogether constitute a `set of malignancy markers` used to distinguish the most malignant canine mammary carcinomas from the others. These genes are also interesting as targets for further investigations and therapy. So far, only two of them were linked with the cancer development.

## Competing interests

The authors declare that they have no competing interests.

## Authors’ contributions

KP: research design, RNA isolation, microarrays analysis, real-time qPCR analysis, primers design, data analysis, manuscript preparation; HM: statistical analysis of microarray experiment; KM: immunohistochemical staining; ID: tumor samples pathological analysis; JM: microarrays printing; TM: research design; MK: research design, immunohistochemical analysis, statistical analysis, manuscript preparation. All authors read and approved the final manuscript.
